# Efficient natural plasmid transformation of *Vibrio natriegens* enables zero-capital molecular biology

**DOI:** 10.1093/pnasnexus/pgad444

**Published:** 2024-02-13

**Authors:** David A Specht, Timothy J Sheppard, Finn Kennedy, Sijin Li, Greeshma Gadikota, Buz Barstow

**Affiliations:** Biological and Environmental Engineering, Cornell University, Ithaca, NY 14853, USA; Biological and Environmental Engineering, Cornell University, Ithaca, NY 14853, USA; Biological and Environmental Engineering, Cornell University, Ithaca, NY 14853, USA; Chemical and Biomolecular Engineering, Cornell University, Ithaca, NY 14853, USA; Civil and Environmental Engineering, Cornell University, Ithaca, NY 14853, USA; Biological and Environmental Engineering, Cornell University, Ithaca, NY 14853, USA

**Keywords:** *Vibrio natriegens*, natural competence, natural transformation, directed evolution, low capital

## Abstract

The fast-growing microbe *Vibrio natriegens* is capable of natural transformation where it draws DNA in from media via an active process under physiological conditions. Using an engineered strain with a genomic copy of the master competence regulator *tfoX* from *Vibrio cholerae* in combination with a new minimal competence media (MCM) that uses acetate as an energy source, we demonstrate naturally competent cells which are created, transformed, and recovered entirely in the same media, without exchange or addition of fresh media. Cells are naturally competent to plasmids, recombination with linear DNA, and cotransformation of both to select for scarless and markerless genomic edits. The entire process is simple and inexpensive, requiring no capital equipment for an entirely room temperature process (zero capital protocol, 10^4^ cfu/μg), or just an incubator (high-efficiency protocol, 10^5−6^ cfu/μg). These cells retain their naturally competent state when frozen and are transformable immediately upon thawing like a typical chemical or electrochemical competent cell. Since the optimized transformation protocol requires only 50 min of hands-on time, and *V. natriegens* grows quickly even on plates, a transformation started at 9 AM yields abundant culturable single colonies by 5 PM. Further, because all stages of transformation occur in the same media, and the process can be arbitrarily scaled in volume, this natural competence strain and media could be ideal for automated directed evolution applications. As a result, naturally competent *V. natriegens* could compete with *Escherichia coli* as an excellent chassis for low-cost and highly scalable synthetic biology.

**Significance Statement**

Bacterial competent cells derived from *Escherichia coli* are a critical component of molecular biology. While these are easy to produce, the process is tedious and requires the tools of a typical biology lab, limiting the democratization of synthetic biology. We demonstrate that the fast-growing microbe *Vibrio natriegens* engineered for natural competence can be used to transform plasmids using a simple process where cells are made competent, transformed, and recovered in the same media without concentration or media exchange, in an optionally entirely room temperature process utilizing no equipment. This work will be of interest to a broad spectrum of researchers including the growing *V. natriegens* community, those interested in directed evolution and automation, and those that lack traditional laboratory equipment.

## Introduction

Over the past decade, the fast-growing microbe *Vibrio natriegens* has attracted significant interest as the next-generation replacement for *Escherichia coli* as a host for synthetic biology and metabolic engineering (1–5). In addition to its extremely fast growth rate, with an optimal doubling time observed to be less than 10 min in rich media (6), *V. natriegens* has a number of advantages as a host for biotechnology, particularly for applications in metabolic engineering (7). Its fast growth rate is enabled by an extremely high rate of both rRNA production (8) and substrate uptake (3), even under anaerobic conditions. It is feedstock flexible, and in particular is capable of growth on low-energy substrates such as formate (9) and acetate (10), and fixes nitrogen under anaerobic conditions (11). Like cloning strains of *E. coli*, it is BSL-1 and is genetically tractable, with a robust selection of promoters, terminators, and ribosomal binding sites (12, 13) and has previously been engineered for production of numerous commercially relevant compounds, including the bioplastic polyhydroxybutyrate (PHB) (14), alanine (3), and 2,3-butanediol (2,3-BDO) (15, 16). Non-sterile seawater can be used as the source of water in growth media without substantial losses in yield, which has implications for sustainable bioproduction (16). Additionally, *Vibrionaceae* appear to be capable of electron uptake from a cathode (17) and thus could be a potential chassis for electromicrobial production (EMP) in which electricity could be used directly to drive microbial metabolism and produce complex bioproducts from simple substrates (e.g. formate, acetate) or carbon fixed via engineered in vivo CO_2_ fixation pathways (18, 19).

While *V. natriegens* is a promising candidate for bioproduction, we contend that another major feature of biotechnological note is its extremely high potential for natural competence, in which cells can uptake intact extracellular DNA under specific environmental conditions. In many models of natural competence in gram negative microbes, for example in Mell et al. (20), natural transformation is often exclusively described as leading to linear ssDNA translocation across the inner membrane prior to homologous recombination. However, this does not appear to be the only possible fate of DNA taken up via natural competence, at least in *V. natriegens*. Natural transformation of intact plasmid DNA was first reported in a *Vibrio* in 1990 (21), reported anecdotally in *V. natriegens* in Simpson et al. (22), and very recently contrasted with linear DNA uptake in a broad study of the genetic underpinnings of natural competency in *V. natriegens* (23).

*Vibrionaceae* in general activate competence under starvation in the presence of chitin found in the shells of crustaceans (24–26). Artificial production of the master competence regulator *tfoX* allows competence to be induced in a more convenient manner, bypassing the need for chitin (but not starvation conditions). This is biotechnologically useful in part because it enables a simple means of conveying large, scarless genomic edits. In a technique called Multiplex Genome Editing by Natural Transformation (MuGENT), originally demonstrated in *Vibrio cholerae*, high rates of cotransformation and subsequent homologous recombination of linear DNA enable the pairing of selectable and nonselectable genomic edits (27). This was later extended to *V. natriegens* via expression of *tfoX* from *V. cholerae*, where transformation rates were found to be as high as 1–10% (14). The method can be further enhanced by the deletion of genes which code for cytoplasmic nucleases which otherwise degrade transforming DNA (28).

While natural competence leading to homologous recombination has been a subject of attention of both biotechnology and fundamental biology, natural competence to dsDNA and plasmids generally, and in *V. natriegens* specifically, remains underexplored. This could be due in part to the existence of robust methods for creating chemically competent and electrocompetent cells in many microbes, including *V. natriegens* (1) which might obviate biotechnological interest in plasmid uptake via natural competence. In this article, we make the case that natural plasmid transformation (NPT), which we treat distinctly from natural competence leading to homologous recombination, is biotechnologically valuable, especially for engineering at huge scales or in low-resource laboratory environments.

**Fig. 1.**
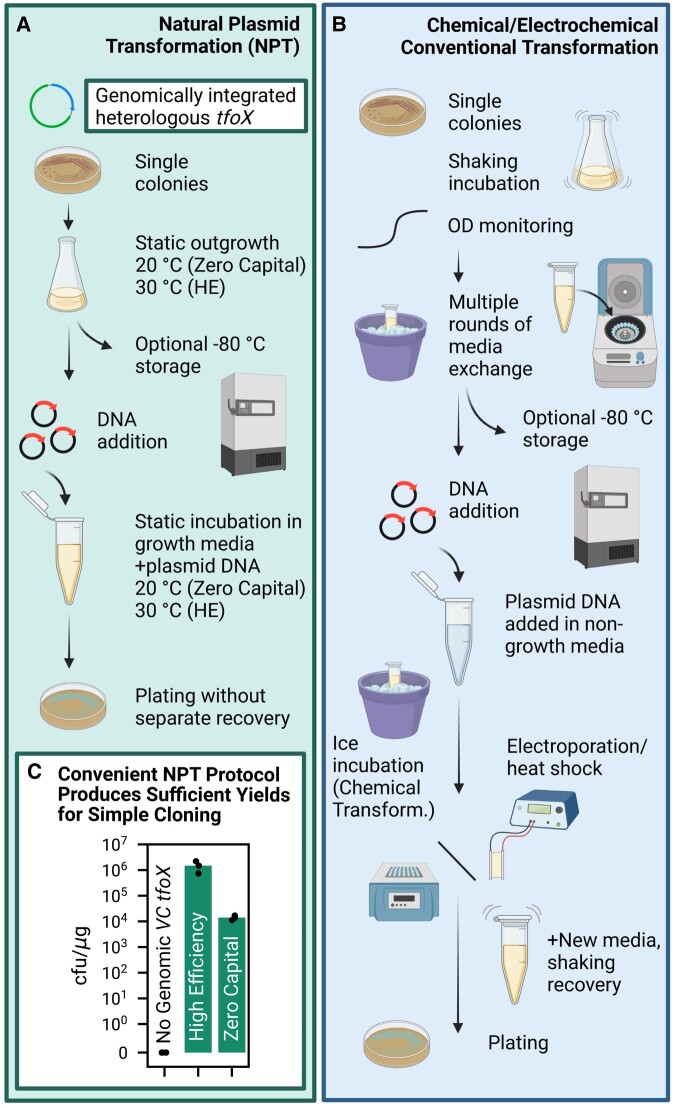
*Vibrio natriegens* genomically engineered for natural competence is transformable via direct addition of plasmid DNA to cells growing in a new Minimal Competence Media (MCM). A) Natural Plasmid Transformation (NPT) is enabled by genomic expression of *tfoX* from *V. cholerae*, which allows for plasmid transformation without media exchange or addition, electroporation/heat shock, or a separate recovery step as is typical for conventional chemical or electrochemical competent cells (B). C) We have developed two protocols, a high-efficiency protocol (HE, 10^6^ cfu/μg), which requires only the use of an incubator and a deep freezer; and a zero capital protocol (10^4^ cfu/μg), which requires no capital equipment at all and can be done entirely at room temperature. Because cells are transformed in their growth media, with no further concentration or media exchange, either protocol can be easily scaled, and the high efficiency transformation can be completed with as little as 50 min of hands-on time when started with frozen NPT competent cells.

In support of the biotechnological case for NPT, we produced a *V. natriegens* strain edited for natural competency, used this strain to develop a pared-down media for growth, preservation, and transformation of cells, and originated an extremely simple protocol for creating and using these cells which optionally uses no capital equipment. Previous biotechnological usage of *V. cholerae tfoX* expression in *V. natriegens* is predicated on use of a plasmid to express it (14, 29). This presents a problem when using *V. natriegens* for cloning of other plasmids, and so we integrated *V. cholerae tfoX* into the genome to obviate this issue. We invented a minimal media which supports both growth and a state of natural competence which is maintained for tens of hours. Cells of our engineered strain are produced and transformed in this singular media, without any exchange, concentration, or separate media recovery, enabling the entire process to be able to be completed with no capital equipment at all, or enhanced with only the use of an incubator and deep freezer (Fig. 1A), which is contrasted with “typical” preparations of chemical or electrochemical competent cells (Fig. 1B). The engineered naturally competent cells can be frozen and conveniently thawed for later use, to our knowledge for the first time by any researcher. We then show that NPT is practically useful, showcasing transformation of plasmids (Fig. 1C), some typical cloning reactions, cotransformation of plasmid and linear PCR product for genomic editing, and then exhibit how the rapidity of both NPT and *V. natriegens* growth can be exploited to do transformation and then isolate single colonies by the end of a typical workday. We establish that natural competence is not just a biological curiosity or just a tool for genomic engineering but allows for a useful third way of plasmid transformation, joining chemical and electrochemical competency.

To be clear, the utility of *V. natriegens* as a cloning tool extends beyond the *V. natriegens* research community. NPT may be of more limited use to a traditional molecular biology lab which could already possess the capital resources for traditional cloning (Fig. 1B). However, low-capital transformation could be a useful for diverse new users including physics or chemistry laboratories seeking to do some limited synthetic biology, low-resource environments like classrooms and labs in the developing world or teaching institutions, iGEM teams, and in high-throughput robotic applications in directed evolution or highly parallelized cloning. The elimination of what may be slight annoyances in typical cloning workflows (using a shaker, keeping cells on ice, etc.) could facilitate scaleup in robotic automation applications where these complexify handling.

Here, we report high-efficiency plasmid uptake in an engineered *V. natriegens* which contains genomically integrated expression of *tfoX* from *Vibrio cholerae* (14, 24, 29), which we believe could be a useful chassis for molecular and synthetic biology research. Ultimately, we demonstrate that this strain can be used for efficient plasmid transformation, using a simplified shared media for competence expression, incubation, and recovery, enabling simple, scalable, low-capital plasmid engineering using *V. natriegens* as a tool.

## Results

### Genomic integration of heterologous *tfoX* creates a *V. natriegens* natural competence strain

It has previously been demonstrated that *Vibrio natriegens* is naturally transformable via plasmid expression of heterologous *tfoX* derived from *V. cholerae* (14). Briefly, in the protocol described by Dalia et al., cells containing pMMB-tfoX (containing Isopropyl β-D-1-thiogalactopyranoside (IPTG)-inducible *V. cholerae tfoX*) are grown up overnight in rich media (LBv2 (1)) with IPTG to overexpress *tfoX*. This dense culture is then diluted 1:100 in an artificial seawater. Transforming DNA (tDNA) is added and cells are incubated statically for 5 h under these starvation conditions, prior to recovery in rich media and plating under selective conditions. While Dalia et al. (14) demonstrate that linear PCR product containing long 3 kB homology arms can be used for genomic editing, in Simpson et al. (22) it is further reported that the same protocol can be used to trigger the uptake of full plasmids in *V. natriegens*.

In initial tests, we confirmed that *V. natriegens* containing either pMMB67EH-tfoX (22) or pST_140_LVL2 cam (from Stukenberg et al. (29), also containing *Vc tfoX*) showed that ectopic expression of *Vc tfoX* via a plasmid can efficiently drive transformation of a second plasmid via natural transformation (NPT). However, because these systems are predicated on plasmid expression of *Vc tfoX*, we sought to create a genomically integrated version which could be used as a natural transformation-based host for molecular biology without interference from a second helper plasmid.

Starting with the type strain (ATCC 14048), we used NT-CRISPR (29) as described to perform a clean deletion of *dns*. NT-CRISPR uses the genomic editing process described in Dalia et al. (14) in combination with a CRISPR counterselection which introduces double-strand breaks at the unedited genomic sequence in order to create scarless genomic edits. *dns* was removed because it encodes an extracellular nuclease which can reduce the efficacy of natural transformation (30) and will likely degrade the quality of plasmid DNA.

We then sought to create a strain with a genomically integrated copy of *Vc tfoX*. Producing such a strain proved to be unexpectedly difficult. We first created plasmid pDS5.29 (which contains IPTG-inducible *Vc tfoX*, Methods) to facilitate integration of linear tDNA containing *Vc tfoX* into the genome. Starting with the *Δdns* strain containing pDS5.29, we used the protocol from Dalia et al. as previously described (14) to simultaneously knock in *camR*, *lacI*, and Ptac-driven *Vc tfoX* in a genomic insertion derived from pST_140_LVL2 cam (29). This insertion, and all subsequent insertions described, are located on chromosome 1, downstream of the gene *glpD* and upstream of *zntB* (Supplementary Fig. S1). Ultimately, however, this worked with extremely low efficiency, and we observed only one working version which retained natural competence after curation of pDS5.29, which contained a broken *lacI* sequence (IPTG inducibility of *tfoX* is lost) which we believe arose spontaneously during production of the tDNA template (Supplementary Fig. S1A). Sequences of both the plasmid used to produce linear tDNA and the entire engineered strain are included in the Supplementary Materials. This strain with the broken *lacI* sequence lacking IPTG inducibility (*Vn* NC1) was validated to do plasmid transformation using the protocol described in Dalia et al. (14) (Supplementary Fig. S2) and used exclusively in the development of a streamlined NPT protocol.

We attempted to restore IPTG inducibility using genomic editing with pDS5.29 as previously described but were not successful. We successfully inserted a different version of the *lacI/Vc tfoX* construct, this time derived from pMMB67EH-tfoX (14) at the same genomic site (*Vn* NC3, Supplementary Fig. S1D) but observed that NPT could not be induced with IPTG in this strain. We then opted to embrace constitutive *tfoX* expression, deleting *lacI* in order to create *Vn* NC4 (Supplementary Fig. S1E), and two versions using the optimized strong constitutive genomic promoter P23 from Wu et al. (13) (*Vn* NC5, NC6, Supplementary Fig. S1F) in lieu of Ptac. However, *Vn* NC4 unexpectedly also did not exhibit NPT, and we were unable to create strains *Vn* NC5 and NC6 because the genomic edit could not be inserted, leading us to speculate that *tfoX* expression from the P23 promoter may be lethal. Ultimately, we opted to continue development using strain *Vn* NC1, although we later revisit the IPTG induction issue in Results Section E.

### Development of Minimal Competence Media (MCM) enables preservation of naturally competent *V. natriegens* without media exchange

We next sought to reduce the protocol described in Dalia et al. (14) such that both outgrowth and transformation could be accomplished in the same media, specifically optimizing for plasmid transformation into our new *Vn* NC1 strain. As previously described, this protocol requires outgrowth in a rich media followed by static incubation under dilute starvation conditions. As the original protocol is already quite simple, the reasoning behind our work was to create a single media which could be used for many rounds of directed engineering, eliminating dilution of dense culture into seawater and making the process simpler to roboticize. We speculated that by combining a simple, suboptimal nutrient (acetate) which is proximal to carbon starvation with the essential components of the artificial seawater mixture we might be able to induce competence in a singular media. We call this eventual mixture of essential salts and acetate minimal competence media (MCM). Further, we found in early development that cells can be readily preserved in the competence state in MCM via flash freezing and revived later for transformation. We sought to determine if these cells could be used as a drop-in replacement for traditional chemically or electrochemically competent cells, transforming an arbitrary plasmid with a pBR322 origin and GFP (green fluorescent protein) expression (pDS5.30, Methods).

In *E. coli*, growth on acetate as a sole carbon and energy source triggers broad catabolite derepression via the cAMP (cyclic adenosine monophosphate) receptor protein (CRP) (31), which is associated with induction of natural competence in diverse species (32) and is a necessary precondition for induction of natural competence in *V. natriegens* (23). Further, it has previously been demonstrated that *V. natriegens* can be grown on acetate as a sole carbon and energy source (3), and we are broadly interested in using acetate as a feedstock because it can be electrochemically produced from CO_2_, which has potential advantages in sustainable bioproduction (33).

**Fig. 2.**
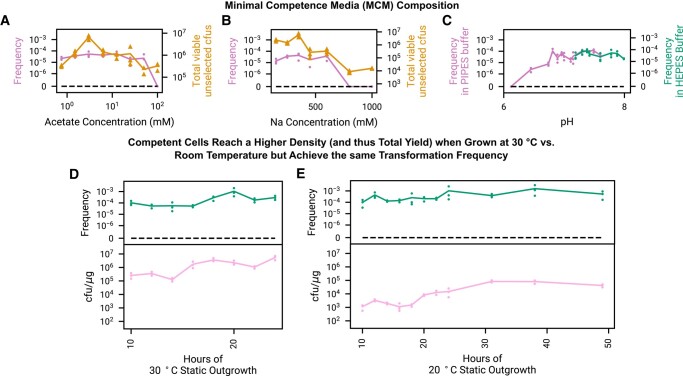
Minimal competence media (MCM) derived from essential seawater components plus acetate is used to both grow out and freeze genomically engineered *V. natriegens* strain NC1 in a state of natural competence. In order to simplify the protocol for eventual directed evolution and automated cloning applications, we developed this singular media which is used to create and transform naturally competent cells. A, B) Using our strain *Vn* NC1 engineered to contain heterologous *V. cholerae tfoX*, we observe that transformation frequency (transformed cfus/untransformed viable cfus) is insensitive to acetate and sodium concentration over a wide range of concentrations, but overall growth (number of viable cfus in 50 μL) is maximized close to 3 mM acetate and 350 mM sodium. C) Transformation is pH dependent and robust in pH ranging from 7 to 8 but falls to zero at lower pH despite growth at pH as low as 6 sufficient to detect natural transformation (Supplementary Fig. S3). In order to center pH in an area with robust transformation and cfu yield, MCM is used at a final pH of 7.4, buffered by HEPES, for all subsequent experiments. D, E) Transformation frequency is relatively insensitive to the period of time that cells are grown out statically prior to transformation, but the overall number of transformed cfus increases as the cells grow in MCM, and cells achieve a much higher maximal density under 30 °C growth. In all of the above figures, cells are flash frozen in glycerol after outgrowth, thawed, incubated statically with 25 ng of DNA for 30 min at 30 °C for transformation, and recovered in LBv2 with shaking at 37 °C for 1 h.

MCM is comprised of a minimal set of essential ions and trace nutrients (Methods) with a low concentration of acetate being used as the sole carbon and energy source. During preliminary experiments, it was determined that acetate concentration and pH were the predominant media determinants of the frequency at which NPT occurs. We did not find a substantial impact due to the concentration of any of the other media components, either in excess or in limiting conditions (including magnesium, nitrogen, phosphorus, and sulfur), nor was it beneficial to add any additional media components which are present at significant levels in natural seawater but inessential for growth (e.g. calcium). Transformation frequency is steady as a function of acetate concentration from 0.75 to 50 mM, but falls off at higher levels (Fig. 2A). The total number of viable cfus are maximized at 3 mM acetate, and this is used for all subsequent experiments. Similarly, a sodium concentration of 350 mM is used in all subsequent experiments as this maximizes the total number of viable cells (Fig. 2B). The natural transformation is highly sensitive to pH, however, as transformation becomes undetectable below pH 6.5 (Fig. 2C), despite the fact that there are a sufficient number of untransformed cells to detect a transformation frequency as low as 10^−6^ (Supplementary Fig. S3). pH is adjusted to 7.4 and buffered using HEPES to center it in the optimal range for all subsequent experiments.

### NPT is streamlined to create an extremely simple transformation protocol with minimal hands-on and total runtime

Briefly, single colonies of strain *Vn* NC1 are used to inoculate 20 mL of MCM, which is grown statically overnight for a period of time at either 30 or 20 °C. After this outgrowth period, 350 μL aliquots are taken, mixed with glycerol, and then flash frozen prior to storage and then subsequent transformation. To transform, cells are thawed, plasmid DNA is added, and cells are incubated statically with the plasmid DNA. In earlier experiments (Fig. 2), cells are recovered at 37 °C in a shaking incubator in rich media for 1 h and then plated.

Transformation is sensitive to the length and temperature of the outgrowth time, with frequency and cfu/μg yield maximized around 18–20 h for outgrowth at 30 °C (Fig. 2D) and around 24–40 h at 20 °C (Fig. 2E). For the case of room temperature outgrowth, the state of natural competence is maintained for at least over a day after the maximal cell density is reached, without substantial loss in transformation frequency or yield after 50 h of outgrowth. In subsequent experiments, cells are collected at 18 h when grown at 30 °C, and at 24 h when grown at 20 °C. Shaking cells during this initial outgrowth stage destroys subsequent competence.

**Fig. 3.**
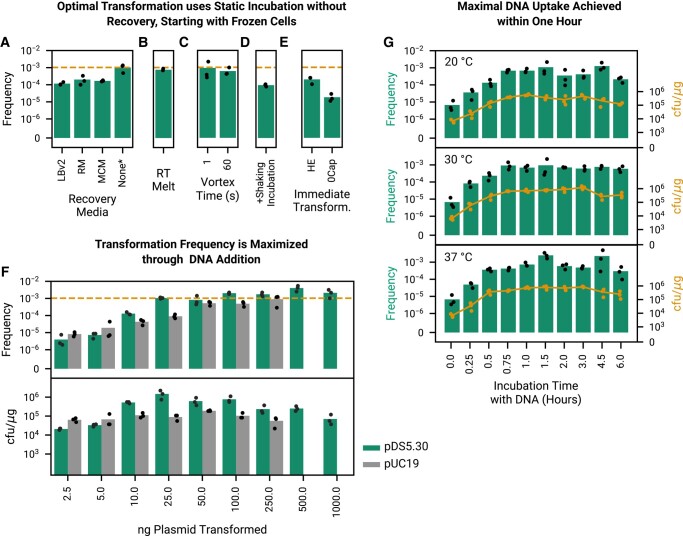
Optimization of transformation of engineered strain *Vn* NC1 shows that natural plasmid transformation (NPT) is extremely fast and remains robust even if many details of working with traditional competent cells are neglected. A) Given a limit of 90 min of hands-on time, allowing cells to incubate for 90 min in MCM yields a higher transformation frequency than a 30-min incubation followed by addition of fresh media (LBv2, recovery media (RM) (1), or MCM) and 60 min of shaking incubation. Because cells are metabolically functional during the active transformation process, they immediately begin to express antibiotic resistance genes, eliminating the value of a separate recovery step. The performance of subsequent experiments are compared to the no recovery condition (dashed line). B, C) Cells can be thawed at room temperature (RT Melt) with no loss in efficiency, and prior to DNA addition can even be vortexed at full speed for up to a minute with minimal losses. D) Cells lose an order of magnitude in transformation efficiency if shaken during incubation rather than being left to take up and express DNA statically. E) We consider full immediate transformation without flash freezing at 30 and 20 °C (starting with cells grown out in 30 or 20 °C, respectively, and proceeding directly after the prior outgrowth step to transformation), corresponding to our HE and zero capital (0Cap) protocols. Interestingly, cells consistently lose approximately an order of magnitude in transformation frequency if they are not flash frozen after they are created. F) In 350 μL of unconcentrated cells in media, yield (transformed cfu per μg of added plasmid DNA) is optimized around 25 ng of added pDS5.30 DNA. Transformation yield with pUC19 is lower. G) In all temperature conditions, optimal transformation efficiency and yield is reached within 45 min of incubation. While eventual transformation yield is sensitive to initial growth temperatures (Fig. 2D and E), incubation with plasmid DNA is accomplished efficiently and comparably from 20–37 °C in a window up to 3 h, with overall yield falling off slightly after that period. Twenty-five nanograms of plasmid DNA is used for experiments in all subplots except F.

We next sought to optimize the protocol for transforming the cells (Fig. 3), finding that because cells remain metabolically active during NPT many aspects of traditional transformation protocols can be omitted. In order to make a transformation protocol which is competitive with chemical transformation in terms of hands-on time, we sought to limit the transformation time to approximately 1.5 h (which would be typical for a chemical transformation with a 30 min incubation and 1 h of recovery, neglecting the other steps). Given a time budget of 1.5 h, we were surprised to find that a 30 min static incubation followed by addition of a recovery media (LBv2, Recovery Media (1), or MCM) under typical recovery conditions (shaking at 37 °C) performs more poorly than simply incubating cells statically for 1.5 h with no recovery step at all and plating cells immediately after incubation (Fig. 3A). We later tested and found that this is similar also for the original Dalia et al. protocol when applied to plasmid transformation, as omitting the recovery step after the 5-h incubation in seawater has a minimal impact on transformation frequency (Supplementary Fig. S2).

Cells are insensitive to being melted at room temperature (Fig. 3B) and are robust and can be vortexed for up to 60 s at maximum speed prior to the addition of plasmid DNA (Fig. 3C). Shaking during incubation reduces transformation frequency by over an order of magnitude but does not destroy it (Fig. 3D).

We next tested the impact of using cells immediately after overnight outgrowth, rather than after −80 °C storage, and found that freezing cells enhances transformation efficiency by almost an order of magnitude (Fig. 3E). We grew out, incubated, and plated each experiment entirely at either 30 or 20 °C. The 20 °C protocol requires no capital equipment at all (no Optical Density (OD) meter, centrifuge, incubator, shaker, freezer, ice machine, heat bath/electroporator, or deep freezer is required), and is the basis of our 0Cap Protocol (Supplementary Note S4), while the 30 °C protocol only requires an incubator. The addition of glycerol prior to flash freezing is not the driver of increased transformation frequency, as the addition of glycerol has no impact when cells are immediately transformed after outgrowth (Supplementary Fig. S4).

We optimized the transformation frequency and yield (cfu per μg of added transforming plasmid DNA) as a function of the amount of added DNA (Fig. 3F) using both pDS5.30 and an arbitrary plasmid commonly used to report transformation efficiency commercially (pUC19). Transformation frequency increases rapidly as up to 25 ng of DNA are added to a standard 460 μL aliquot of cells, with marginal increases in total frequency as up to several hundred nanograms are added, with cfu/μg yield maximized around 50–250 ng. pDS5.30 consistently provides a higher yield at these concentrations.

Transformation frequency and yield is insensitive to incubation temperatures ranging from 20 to 37 °C, increasing with incubation time until plateauing around 45 min (Fig. 3G). Transformation in which cells are plated immediately after DNA addition with no incubation at all results in a surprisingly high transformation frequency (≈ 10^−5^), perhaps indicating that some of this activity may be occurring on the plate as dilutions dry. Incubation on ice results in no transformants (not shown). This result, in combination with the prior result finding that transformation efficiency is not improved by the addition of recovery media, indicates that cells begin expressing antibiotic resistance genes from the plasmid immediately upon uptake during static incubation in MCM, unlike with traditional chemically competent cells which are inactive when incubated on ice.

High energy food sources like sugars inhibit NPT in a singular media where there is no subsequent dilution or media exchange step, likely due to carbon catabolite repression impacts on natural competence. Using the minimal MCM components, and a diverse array of potential metabolites in lieu of acetate at the same 3 mM concentration (glucose, sucrose, gluconate, formate, pyruvate, sorbitol, and tryptone), we sought to see if there might be options other than acetate as a carbon/energy source in MCM. We found that pyruvate is the next-best inducer of natural transformation (Supplementary Fig. S5), which is competent at a frequency on the order of 10^−4^. A mixture of 10% LBv2 and 90% artificial seawater also exhibited transformation, although at a low frequency on the order of 10^−6^. Replication of the creation of competent cells using pyruvate MCM and 10% LBv2 with flash freezing and −80 °C storage shows improved transformation frequency (Supplementary Fig. S6), as was the case for acetate MCM.

### Naturally competent *V. natriegens* is an effective tool for plasmid cloning

**Fig. 4.**
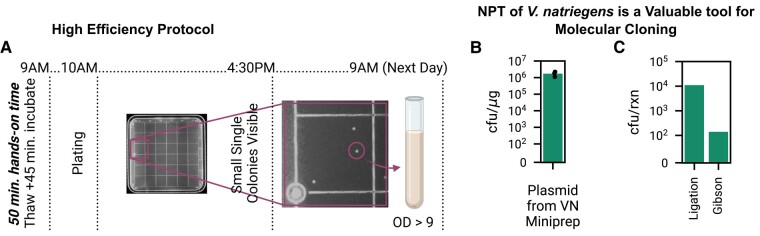
NPT of genomically engineered *V. natriegens* produces culturable single colonies within a standard workday, empowering rapid cloning. A) In our rapid, HE (requiring only an incubator and deep freezer), *V. natriegens* transformation and growth is so fast that single colonies are visible (≈ 0.7 mm) and can be picked within a standard workday when transformation is done first thing in the morning. B) Plasmids miniprepped from *V. natriegens* are transformable back into *V. natriegens* with the same yield as those produced from *E. coli* DH5*α*. C) Arbitrary, common molecular biology reactions (a ligation via Kinase, Ligase, and Dpn1 (KLD), deleting GFP expression from pDS5.30; and a Gibson assembly, inserting GFP expression into a pUC19 backbone) can be transformed directly into the natural competence strain of *V. natriegens* without any additional cleaning steps, yielding 11,000 and 140 transformants, respectively, from 2 μL of reaction product.

Transformation of frozen competent cells through isolation of single colonies is possible within a standard workday due to the combination of rapid *V. natriegens* growth and the fact that a 45-min incubation is sufficient to maximize NPT transformation frequency and yield (Fig. 4G). As a simple demonstration of this capability, we began a transformation at 9 AM (Fig. 4A). NPT competent cells were removed from the deep freezer and then thawed on the benchtop for ≈ 5 min. Transforming plasmid DNA was then added and cells were incubated for 45 min at 30 °C, and then immediately plated (37 °C) on pre-warmed LBv2 agar plates using plating beads at around 10 AM. By 3:30 PM, extremely small single colonies were visible, which we then imaged at 4:30 PM and used a single one to inoculate a culture tube of LBv2. By 9 AM on the following day, that overnight culture yielded an OD of 9.2 in 3 mL of media.

The ability to get to the single colony stage within a standard workday is significant and gives a putative cloning strain of *V. natriegens* an edge in utility over *E. coli*. Upon plating, transformants of *E. coli* cloning strains will not be visible to the naked eye after 6 h of growth. As a result, outgrowth of colonies is typically carried out overnight. This means that, depending on timing, many protocols which typically take two days to complete can be done in one if started in the morning.

Plasmids can be readily extracted from *V. natriegens* using standard miniprep kits developed for use with *E. coli*. These plasmids can then be used to transform *V. natriegens* with the same yield as plasmids derived from *E. coli* DH5*α* (Fig. 4B).

We next demonstrated that two arbitrary molecular biology reactions can be transformed directly into *V. natriegens* NPT competent cells (Fig. 4C). We used KLD, an enzyme mixture produced by NEB which is used to circularize linear PCR fragments. This product consists of kinase, ligase, and dpn1 optimized to ligate PCR product and remove confounding template plasmid DNA in a single reaction. We used PCR to delete the GFP expression sequence from pDS5.30 and KLD for the subsequent ligation to create new plasmid pDS5.44. We then directly transformed 2 μL of this reaction product (the standard volume recommended by NEB for a transformation with *E. coli*) into our *Vn* NC1 competent cells via NPT, yielding 11,000 transformed cfus.

Similarly, we used Gibson assembly (via NEB Hifi) to insert a GFP expression sequence into pUC19, producing new plasmid pDS5.43. NPT transformation of 2 μL of Gibson assembly reaction product into strain *Vn* NC1 produced a yield of 140 transformed cfus.

### Cotransformation of plasmid and linear DNA enables scarless genomic editing

Because natural transformation is a highly non-Poisson process such that a DNA uptake event is strongly correlated with additional uptake events (34), it is expected that there are many instances of cotransformation where several distinct DNA molecules are taken up by the same cell. Therefore, in the presence of mixed selectable and nonselectable genetic material, if a cell takes up selectable material, there is an increased likelihood that unselected material was taken up simultaneously. The high rate of cotransformation is used in MuGENT (14, 27), where selectable and nonselectable genomic edits are paired in order to rapidly incorporate genomic edits which are not directly selectable.

**Fig. 5.**
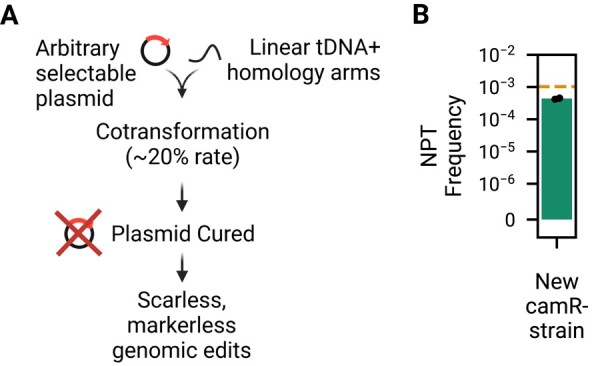
Cotransformation of plasmids and linear DNA enables rapid scarless and markerless genomic edits of genomically engineered *V. natriegens*. A) As in MuGENT (28), unselected genomic edits can be paired with selectable markers due to a high rate of natural competence cotransformation. By using a plasmid to convey selection, the resulting strain can be easily cured of the plasmid without leaving a selectable marker behind in the genome. B) We use this cotransformation to remove *camR* from the strain used throughout this study and the resulting strain *Vn* NC7 retains natural competence.

We reasoned that it should be similarly possible to cotransform both selectable plasmid DNA and arbitrary linear DNA at an increased rate for straightforward genomic editing in *V. natriegens* (Fig. 5A). In all previous experiments, the natural competence strain of *V. natriegens* still contained the chloramphenicol resistance gene *camR*. This is unnecessary and undesirable for subsequent cloning which might utilize chloramphenicol, and thus we sought to delete it. In order to test our cotransformation hypothesis, we attempted to cotransform an arbitrary plasmid (pDS5.30, which contains kanamycin resistance) and tDNA for homologous recombination-based deletion of *camR*, derived from plasmid pDS5.45. Using 400 ng of transforming DNA with 3 kB homology arms, paired with 25 ng of pDS5.30, we cotransformed these and determined that 22% of cells received the desired edit (8 out of 36 colonies tested for simultaneous loss of genomic chloramphenicol resistance and gain of plasmid-based kanamycin resistance), indicating a high rate of cotransformation of the *camR* deletion along with plasmid uptake. Cells were then easily cured of pDS5.30 and we verified with whole-genome sequencing that the resulting strain had the expected sequence and remained naturally competent, although with reduced transformation frequency (Fig. 5B).

### RT-qPCR establishes upper and lower limits of mRNA *Vc tfoX* expression sufficient for natural competence induction

Next, we again sought to correct the missing *LacI* sequence present in both strains *Vn* NC1 and NC7, attempting to restore 67 base pairs at the end of the *LacI* sequence (see pDS5.59 in Supplementary Information). Starting with strain NC1, and using the cotransformation protocol described in the previous subsection, we attempted to simultaneously knock out *CamR* and restore the missing *LacI* base pairs. However, the strain appeared to be highly resistant to this edit, with only a single colony out of approximately 40 tested appearing to contain *camR* loss (despite a high 20% success rate producing *Vn* NC7 from NC1 in a very similar edit). The resulting strain, *Vn* NC8 (Supplementary Fig. S1C), was Sanger sequenced to confirm *lacI* restoration of the missing base pairs, however subsequent whole-genome sequencing revealed a new, completely novel deletion of 233 base pairs within the *lacI* sequence. Prior to receiving the sequencing results, we were disappointed to find that this new strain continued to lack IPTG inducibility (Supplementary Fig. S7), with constant transformation frequency at all levels of IPTG induction, comparable to that of *Vn* NC7.

We sought to understand the root cause of our challenges in cloning IPTG inducible *Vc tfoX* expression. In the new strain *Vn* NC8, we observed a curious toxicity stemming from IPTG addition, where the strain exhibited lowered total yield (Supplementary Fig. S9A) due to reduced total survival as a function of added IPTG (Supplementary Fig. S9B). Anecdotally, in prior tests of the use of our finalized protocol optimized for our genomically integrated expression of *tfoX* on the *Δdns* strain containing the plasmid pMMB67EH-tfoX (22), we also found that while IPTG induction worked as expected (Supplementary Fig. S8), it also appeared to reduce total survival in MCM at 100 and 200 μM IPTG, regardless of plasmid uptake. In Supplementary Fig. S8, pMMB67EH-tfoX is induced with 50 μM IPTG, half of what is typical. We had assumed that this must be due to lethal *tfoX* overexpression under stressful natural competence conditions, although this now appears to be incorrect. The reason for this IPTG toxicity remains unclear and is discussed further in Supplementary Note S2.

Disappointed that inducibility could not be restored in our NPT strains, we used RT-qPCR in order to shed additional light on relative *tfoX* expression levels in order to better understand our working versions. We extracted RNA from each strain grown out in competence-inducing MCM conditions and measured *tfoX* expression relative to expression of arbitrary gene *gyrB* (Supplementary Fig. S10). As expected, the *Δdns* strain containing pMMB67EH-tfoX (14) shows unambiguous inducibility with IPTG (Supplementary Fig. S10A, using only 50 μM IPTG for induction), with a dynamic range spanning two orders of magnitude. Strain *Vn* NC1, with genomically integrated *tfoX*, exhibits substanially lower *tfoX* mRNA expression (83.6% lower than pMMB67EH-tfoX maximum expression, Supplementary Fig. S10B). Strain *Vn* NC7 has still lower expression (94% lower than the pMMB67EH-tfoX maximum, and 63.5% lower than *Vn* NC1, Supplementary Fig. S10C). Despite apparent IPTG toxicity (Supplementary Fig. S9), *Vn* NC8 does not appear to exhibit any significant changes in *tfoX* expression as a function of IPTG addition, as could be expected given the large deletion, but furthering the mystery of apparent toxicity (Supplementary Note S2). Across the spectrum of IPTG conditions, *Vn* NC8 expresses *tfoX* mRNA at levels comparable to *Vn* NC7.

Despite detectable circuit leak from *tfoX* expression by pMMB67EH-tfoX (Supplementary Fig. S10A) in the absence of IPTG, it is sufficiently tolerated such that there are no detectable escaped transformants (Supplementary Fig. S8). This establishes an upper ceiling of *tfoX* mRNA expression that can be tolerated in the OFF condition without unwanted natural competence expression. At the high end of expression, there appears to be a floor for *tfoX* expression sufficient to maximize NPT frequency, as despite the fact that maximal *tfoX* expression by pMMB67EH-tfoX does not increase NPT frequency beyond what is observed with *Vn* NC1 (Supplementary Fig. S8, relative to the orange line). However, differences in *Vn* NC1 and NC7 transformation frequency (Fig. 5B, relative to the orange line) are probably due to reduced *tfoX* transcriptional expression in *Vn* NC7 relative to *Vn* NC1.

## Discussion

In this work, we have shown that a genomically engineered strain of *V. natriegens* created specifically for enhanced natural competence can be used as an effective chassis for low-cost and low-capital plasmid engineering. Natural plasmid transformation (NPT), using our strain and specific transformation media, allows for alternate growth and high-efficiency transformation without exchange of media. Further, cells can be flash frozen and stored in this naturally competent state. Plasmids can be cotransformed with linear DNA for genomic editing, enabling diverse scarless genomic edits without requiring additional genomic insertions (28) or additional plasmid engineering for CRISPR counterselection (29). Due to the low-resource intensity and yet high efficiency of the transformation protocol, this work supports the idea that *V. natriegens* is a strong candidate next-generation molecular biology workhorse, especially for low-resource laboratory environments. In particular, we believe that a chassis which readily grows and can be edited entirely at room temperature, using no capital equipment, could be a radical new resource for the democratization of synthetic biology, especially in education.

NPT is a curious third alternative to chemical or electrochemical transformation of plasmids in *V. natriegens*. Many microbes are capable of a state of natural competence under the right conditions (35), and many more are likely to be as specific environmental triggers are discovered. Even *E. coli*, the current molecular biology workhorse, is capable of natural transformation of plasmids while on solid media (36, 37) independent of the type IV pilus (38), although the efficiency and protocol make it impractical for routine cloning. In general, natural competence in bacteria is controlled by an eclectic set of diverse environmental and physiological conditions. While natural transformation is used extensively as a tool to modify diverse microbial genomes, few microbes have previously been engineered specifically for enhanced natural competence, most notably *B. subtilis* (39, 40) and *V. cholerae* (27). *B. subtilis* is a poor substitute for *E. coli* due to its evolutionary distance, preference for multimeric plasmids, and poor diversity of plasmids mutually compatible with *E. coli* (41). To our knowledge, there are no demonstrations of cells in a naturally competent state with comparable general utility to traditional frozen *E. coli*-based chemical competent cells. In Simpson et al., the reported transformation frequency into *V. natriegens* is substantially higher ($\approx 10^{-2}$ (22)) than what we observe, which is more in line with what is observed in Shin et al. ($\approx 10^{-3}$ (23)), indicating that there may yet be additional variables to manipulate to further improve performance.

The uptake of intact circular, double-stranded DNA runs counter to models of natural transformation in gram negative microbes which are predicated on solely ssDNA translocation across the inner membrane prior to homologous recombination, e.g. as described in Mell et al. (20). Retraction of type IV competence pili which bind dsDNA draws it to the cell surface and mediates DNA internalization, driving natural competence (42). dsDNA could then be taken up by putative membrane elements which pass dsDNA through the inner membranes, as appears to be the case for limited natural transformation of *E. coli* on solid media (43). Alternatively, it is also possible that two ssDNA plasmid monomers are taken up individually and reconstituted inside the cell, as is the case in *S. pneumoniae* (44). Transformation of plasmid monomers could be used to differentiate between such single- and double-hit kinetics, as is done in Sun et al. (45). The fact that our system depends on *tfoX* expression (Fig. 1C), which is an essential trigger for type IV pilus-based DNA uptake and homologous recombination (14, 27), indicates an effective comingling of systems for both double and single-stranded DNA uptake. Very recent work (23) identifies a new membrane protein which appears to be essential for genomic linear tDNA integration but not plasmid DNA uptake. Our observation that the frequency of transformation is significantly enhanced by flash freezing also indicates that there could be some role of physical trauma to the cell membrane which either promotes competence gene expression or directly facilitates DNA uptake.

Inability to restore inducibility of our genomic *tfoX* construct has been a source of immense frustration. Ultimately, it will be important to be able to turn natural competence off, as constitutive expression of genes driving homologous recombination could promote genomic instability. Additionally, as reported in Shin et al. (23), *tfoX* overexpression causes increased sensitivity to reactive oxygen species. As there is a ceiling on *tfoX* overexpression efficacy (Supplementary Fig. S8), there is likely not any advantage to further overexpressing it. Therefore, we believe that we have largely exhausted the improvement that could be done by optimizing *tfoX* expression and will be pursuing alternative routes toward dysregulating natural competence. However, in future work we will pursue alternative inducible promoters to see if inducibility of competence can be achieved by some different means.

NPT can be efficiently accomplished with 50 min of hands-on time, less than half of the time required for chemically competent cells (Fig. 4A), and requires neither a heat shock nor electroporation. Additionally, NPT competent cell preparation is extremely simple, requiring no wash steps and functioning within a wide time window which does not require monitoring of OD (Fig. 2D). It is a dynamic process by which cells actively take up DNA in a physiologically controlled process at temperatures relevant for growth, as opposed to chemical transformation which occurs via diffusion while cells are maintained on ice. The fact that cells remain metabolically active under these conditions likely explains why a separate recovery step is not required in order to express antibiotic resistance genes (Fig. 3A). In total, this simplicity is automation friendly and enables some unique applications. For example, it would be possible to independently transform collections of many clonally isolated individuals, at room temperature and with no separate chilling or shaking steps, in 96-well plates in MCM.

The extremely high rate of natural transformation in *V. natriegens* with *tfoX* expression makes NPT practically relevant in routine cloning applications (Fig. 4B and C). However, as a matter of yield in transformed cfu per added microgram of DNA, it is not yet competitive with commercial strains of cloning *E. coli*. Yields are lower than practically similar one-step chemical transformation methods in *E. coli*, which also do not require media exchange or heat shock and achieve a transformation efficiency of ≈ 10^7^–10^8^ cfu/μg (46). However, because natural transformation is an active process by which cells take up DNA under metabolically functional conditions, it is likely that the rate of natural transformation can be increased through engineering as has been done previously in *V. cholerae* (27), either by increasing the frequency of competence pili production or retraction, or by making the process insensitive to growth in rich media.

Aside from general cloning usage, the fact that cells can be grown, transformed, and subsequently recovered in the same media all at the same temperature without media exchange could have powerful advantages in continuous evolution. For example, in multiplexed automated genome engineering (MAGE) (47), DNA for recombineering is delivered via electroporation and the process is thus bottlenecked by transformation efficiency (48). Especially given the feedstock flexibility and extremely high growth rate of *V. natriegens*, NPT of nonreplicative plasmids and directed evolution could represent a potent combination. While *E. coli* can also be grown and transformed in a shared media (46), cells must be chilled during transformation and recovered under growth conditions prior to antibiotic selection.

Ultimately, one must ask the question of whether researchers would actually switch to a replacement for *E. coli* for molecular cloning. While *V. natriegens* has a multitude of advantages and could serve as a drop-in substitute in many applications, for example in protein production (49) and simple DNA assembly (Fig. 4C), dominance of classic *E. coli* strains like DH5*α* is due to their proven reliability over decades. However, the reliance on the hardware of a textbook biology lab limits the reach of synthetic biology. NPT via *V. natriegens* which enables low-resource end users to use the tools of molecular biology will push the field forward and further the democratization of synthetic biology.

## Materials and methods

### Working with *V. natriegens*

In general, growth of *V. natriegens* was done in LBv2 liquid media or LBv2 agar plates (1). When necessary, antibiotics were used in both solid and liquid culture at a final concentration of 200 μg/mL (kanamycin), 2 μg/mL (chloramphenicol), and 10 μg/mL (carbenicillin, see Supplementary Note S1). In instances where it was necessary to transform *V. natriegens* via conventional means, cells were made electrocompetent using the protocol as described in Weinstock et al. (1). Glycerol stocks were created by mixing cells in late exponential growth (≈ OD 1) at a ratio of 3:1 with 60% glycerol prior to storage in a −80 °C freezer. In instances where artificial seawater media was used, we filter sterilized 28 g/L of Instant Ocean Sea Salt in deionized water.

### Genomic editing of *V. natriegens*

A *dns* knockout of the *V. natriegens* wild type strain (ATCC 14,048) was created using NT-CRISPR, using the protocol as described in Stukenberg et al. (29). Briefly, this protocol uses natural transformation of linear tDNA with flanking 3 kB homology arms (14) to make genomic edits, followed by CRISPR-based counterselection which introduces double-strand breaks selecting against the original, unedited sequence. The requisite 3 kB homology arms amplified from *V. natriegens* WT genomic DNA as a template were assembled in pUC19 via Gibson assembly (NEBuilder HiFi DNA Assembly), creating pDS5.13. This plasmid was then used as a template to create the tDNA needed for genomic editing via PCR amplification using DNS_Upstream_F/DNS_Downstream_R. CRISPR counterselection was accomplished using the spG Cas9 NT-CRISPR plasmid (pST_140_LVL2 cam, Addgene 179334) with spacer sequence tgcactatccagtgccgccg (pDS5.17), as described in Stukenberg et al., using annealed primers dns_gRNA_F/dns_gRNA_R to replace a sfGFP dropout fragment with the gRNA spacer sequence.

The annotated sequence of this plasmid, and all plasmids in this work, along with primer binding sites illustrating how they are used in assembly, can be found in SupplementaryInformation.xlsx via links hosted via Benchling. Genbank exports of the Benchling files are also provided.

We then inserted the *tfoX/lacI/camR* construct from Stukenberg et al. (29) into the *Δdns V. natriegens* strain. In order to accomplish this, we created a second helper plasmid (pDS5.29) containing *tfoX* and GFP to aid in eventual curation. pDS5.29 is created using the origin, kanR, and GFP expression sequences from pEvolvR-enCas9-PolI3M-TBD (50) and the lacI/tfoX expression sequence from Stukenberg et al. pST_140_LVL2_cam using Gibson assembly with primers 528_BB_F/ 529_BB_CORRECTED_R, GFP_Liftout_F/ GFP_Liftout_R and 529_Ins_CORRECTED_F/ 528_Ins_R.

Using this helper plasmid and tDNA derived from pDS5.27 (containing the insertion and homology arms), we inserted the construct into the genome following the protocol for natural transformation from Dalia et al. (14). Cells were plated for chloramphenicol resistance, and verification of the insertion at the expected location was confirmed by colony PCR. As in NT-CRISPR, cells were then cured of the helper plasmid via 37 °C antibiotic-free growth in LBv2 for 6 h, plating a 10^−7^ dilution on LBv2 agar plates, and then striking colonies onto kanamycin plates in order to verify helper plasmid loss.

### NPT protocol development

Plasmid pDS5.30 was created explicitly as a test case for optimization of the NPT protocol. It is derived from the plasmid pEvolvR-enCas9-PolI3M-TBD (50), edited to remove the EvolvR system which was not utilized in this work. The plasmid contains GFP expression which makes it easy to determine that transformants received the plasmid and are not spontaneous abx-resistant mutants.

In all iterations, cells of the natural competence strain or the negative control were first struck out from a glycerol stock onto LBv2 plates for single colonies. On the subsequent day, a single colony was used to inoculate 20 mL of media in 100 mL flasks to create cells with a state of natural competence. In instances where cells are preserved in the −80 °C freezer, 350 μL of overnight culture is added to 110 μL of 60% glycerol, mixed by pipetting up and down, placed on ice for several minutes, and then flash frozen in liquid nitrogen. In early iterations, cells from the freezer are thawed on ice (Figs. 2 and 3A), while in subsequent experiments cells are thawed on the benchtop. In instances where cells are used for immediate transformation without freezing, DNA is directly added to 350 μL of overnight culture, except in Supplementary Fig. S4 where 110 μL of 60% glycerol is also added. Except in cases where the amount of DNA is explicitly changed (Fig. 3F), 25 ng of plasmid DNA is used for all NPT transformations.

Cells are then incubated in the presence of the plasmid tDNA. This is done statically, with the exception of Fig. 3D, for a period of time ranging from 0 to 6 h, at either 30 °C or room temperature. In early iterations (Fig. 2), 1 mL of LBv2 is added and cells are placed in a shaker at 37 °C for recovery. After it became clear that there was limited benefit from the addition of recovery media (Fig. 3A, discussed in the main text), cells are plated immediately after incubation, diluting as appropriate in order to calculate transformation efficiencies. Plated cells are grown out at 37 °C, except for in Fig. 3E 20 °C which is a demonstration of the fully room temperature protocol.

In its final manifestation, MCM consists of: 9 mM HEPES, 3 mM sodium acetate, 1.9 mM ammonium chloride, 1.6 mM potassium phosphate, 7 mM potassium chloride, 1 mM magnesium sulfate, 31 mM magnesium chloride, and 350 mM sodium chloride. In order to prevent precipitation, 1 mL of undilute hydrochloric acid is used to lower the pH of 900 mL of deionized water prior to adding the media components and water to a total volume of 1 L. The final mixture is then adjusted upwards to pH 7.4 using 1 M sodium hydroxide and sterile filtered. The media will precipitate if autoclaved. This recipe is used for all experiments in included figures except Fig. 2A–C, indicated in pink, where 10 mM PIPES is used in lieu of HEPES and the pH is adjusted to 7, and in Supplementary Figs. S5 and S6, where various carbon/energy compounds are used in lieu of acetate, as indicated.

We have written instructions for NPT in our natural competence strain, describing both the high speed/efficiency and low-capital transformations, as protocols in Supplementary Notes S3 and S4, respectively.

With the exception of Fig. 3F where indicated in gray, all plasmid transformations were done using pDS5.30, a plasmid with a pBR322 origin which expresses kanamycin resistance and GFP.

### Development of NPT as a tool for cloning

In order to test the utility of NPT and *V. natriegens* as a host for cloning, we designed two arbitrary Gibson assembly and KLD reactions, creating final plasmids pDS5.43 and pDS5.44, respectively. The requisite PCRs were completed using NEB Hot Start Q5. PCR product for Gibson assembly was cleaned using a Zymo Clean & Concentrator kit and then used in a NEBuilder HiFi DNA Assembly reaction as described by the manufacturer.

In the KLD reaction (NEB KLD Enzyme Mix), pDS5.30 is PCR amplified using primers TransientKan_F and DelGFP_R in order to excise the GFP sequence from pDS5.30, producing plasmid pDS5.44 and an easy-to-visualize change from green to white cells. This PCR product is used directly in the KLD reaction without further cleaning, per the manufacturer’s instruction. In both, 2 μL of reaction product is added directly to the competent cells in media, just as with plasmid DNA in the previously described NPT protocol.

Miniprep extraction of plasmid DNA from *V. natriegens* was accomplished using the E.Z.N.A Plasmid DNA Mini Kit I produced by Omega Bio-Tek, following the manufacturer’s instructions.

For the demonstration of producing single colonies within a standard workday, colonies are imaged using an Azure Biosystems Gel Imaging System. The image contrast is altered in order to highlight the presence of colonies and facilitate measurement of their size.

### Cotransformation of plasmid DNA with linear tDNA for genomic editing

tDNA with 3 kB homology arms (as described in Dalia et al. (14)), designed for the deletion of *camR* which was previously inserted, was created by assembly of plasmid pDS5.45 via Gibson assembly, from which tDNA was amplified with PCR using primers CamRtDNALift_F and CamRtDNALift_R and column purified. Cotransformation of plasmid DNA and linear tDNA with homology arms for genomic editing was done using the described protocol for plasmid transformation in our natural competence strain. Twenty-five nanograms of pDS5.30 was cotransformed with 400 ng of linear tDNA. Once plated, we streaked 36 arbitrary colonies onto chloramphenicol plates in order to estimate the frequency of genomic editing. Deletion of *camR* was additionally verified by colony PCR and Sanger sequencing. Whole-genome sequencing was done by Plasmidsaurus.

We attempted to use the same procedure in order to restore the broken *lacI* sequence present in strains *Vn* NC1 and NC7. We used Gibson assembly to insert the *lacI* sequence from pST_140_LVL2 cam into pDS5.27 but with a changed compositional context (removing *camR* but retaining the *camR* terminator sequence), producing plasmid pDS5.59. As with creation of linear tDNA from pDS5.27, we used primers _5.25_Liftout_F/R to amplify tDNA from this plasmid template and used this in conjunction with pDS5.30 for cotransformation as previously described (400 ng linear DNA, 25 ng pDS5.30 plasmid). As discussed in the main text, the only version which received an edit deleting *camR* also contained a substantial new defect in the transferred *lacI* sequence.

### RT-qPCR to measure relative *Vc tfox* mRNA concentration

For each condition, 20 mL of MCM plus the appropriate amount of IPTG was inoculated with a single colony of the specified strain and incubated at 30 °C for 18 h. Samples were then either prepared directly after for RNA extraction or were frozen at −80 °C until needed before being thawed on ice.

Due to the low density of culture growth in MCM, samples were centrifuged for 10 min at 4 °C in 15 mL tubes order to concentrate them. All but 2 mL of media are aspirated from the tube and is then centrifuged again for an additional 2 min at 4 °C in a microcentrifuge tube. All but 200 μL of media are then aspirated from the tube and the remainder is resuspended by pipetting.

The Zymo Direct-Zol RNA Miniprep Plus Kit was then used to purify 100 μL of the remaining fluid into 50 μL of concentrated RNA. Forty-three microliters of this RNA is then digested using 4U of DNase in a 50 μL reaction for an initial 30-min reaction incubated at 37 °C, then an additional 4 U of DNase are added for a second 30-min reaction at 37 °C. One microliter of 0.5 M EDTA is then added and the mixture is heat inactivated at 75 °C for 10 min.

The resulting solution was then cleaned using the RNA Clean and Concentrate-5 Kit from Zymo and eluted into 40 μL of ddH2O. Concentrations for RNA samples were taken on a Qubit Fluorometer. Any samples with RNA concentrations too low were reconcentrated into 15 μL of solution using the same kit.

In order to prepare samples for development of the qPCR standard curves, segments of *tfoX* and *gyrB* were PCR amplified using a cPCR protocol with OneTaq HS 2X MM with Standard Buffer on single colonies from the *Vn* NC7 strain. Resulting samples were cleaned using the DNA Clean and Concentrator-5 from Zymo. Concentration measures were taken on the Nanodrop Fluorometer.

For each qPCR run, the LunaUniversal One-Step MasterMix was utilized in the QuantStudio 7 Pro. Standard curves were generated using *tfoX* and *gyrB* standards at dilutions ranging from 1:10 to 1:100,000. Two microliters of each of these diluted standards were combined with the recommended quantities of the Luna Mix in triplicate.

For each test sample, 5 ng of total RNA were added to the recommended quantities of Luna Mix in triplicate, as well as a noRT negative control where the reverse transcriptase was removed, a positive control to measure for baseline expression of the *gyrB* control gene, and a *gyrB* noRT control containing the *gyrB* primers without reverse transcriptase. All reactions had a final volume of 20 μL.

## Supplementary Material

pgad444_Supplementary_Data

## Data Availability

All underlying experimental and sequencing data are hosted at the Barstow Lab GitHub: https://github.com/barstowlab/VnatNC1. Strains are available upon request and will be hosted by Addgene with IDs 215355 (*V. natriegens* NC1) and 21356 (*V. natriegens* NC7).
