# Supplementary material for: Efficient natural plasmid transformation of Vibrio natriegens enables zero-capital molecular biology
Source: PNAS Nexus. 2024 Feb 13;3(2):pgad444. doi: 10.1093/pnasnexus/pgad444 (PMC10863642; doi:10.1093/pnasnexus/pgad444)
Supplement: pgad444_Supplementary_Data [file pgad444_supplementary_data.zip › PNASNEXUS-PNASNEXUS-2023-00682R-s01.pdf]

Supplementary Materials for: Efficient Natural Plasmid Transformation of *Vibrio natriegens* Enables Zero-capital Molecular Biology

David A. Specht,† Timothy J. Sheppard,† Finn Kennedy,† Sijin Li,‡ Greeshma Gadikota,¶ and Buz Barstow*,†

†*Cornell University, Biological and Environmental Engineering, Ithaca, NY 14853*

‡*Cornell University, Chemical and Biomolecular Engineering, Ithaca, NY 14853*

¶*Cornell University, Civil and Environmental Engineering, Ithaca, NY 14853*

E-mail: bmb35@cornell.edu

Note S1 Ampicillin vs. carbenicillin sensitivity in *V. natriegens*

While ampicillin and carbenicillin are treated interchangeably when used for *E. coli* selection, we have observed that in *V. natriegens* carbenicillin exhibits dramatically stronger selection on solid media than ampicillin does. In our tests, concentrations as low as 2 μg/mL of carbenicillin can be sufficient for counterselection, while there are colonies which escape ampicillin selection at concentrations as high as 50 μg/mL in freshly made plates. Ultimately, we use carbenicillin at a concentration of 10 μg/mL for selection of pUC19 recipients (Methods). A prior review of methods in *V. natriegens* antibiotic concentrations[5] shows that there may not have been contrasting usage of these two antibiotics in

prior studies.

Further, however, we observe that *V. natriegens* grown out under robust conditions (outgrowth in unselective rich liquid or solid media) can quickly develop tolerance to carbenicillin selection on plates. While cells from a weakened state (glycerol stocks or cells grown out in our described MCM protocol) will die on plates with 10 μg/mL carbenicillin, cells which are taken from rich liquid media or streaked from single colonies will require stronger plate counterselection. Essentially, use carbenicillin/ampicillin plate selection with caution. We do not observe this issue in antibiotic selection in liquid culture.

We observe this phenomenon in the ATCC type strain and thus this is a general problem when working with *V. natriegens* and not the result of our genomic editing. In further development of *V. natriegens* as a drop-in replacement for *E. coli*, it may be advantageous to edit strains for enhanced sensitivity to beta lactam antibiotics, for example by deleting genomic beta lactamases as identified in Lee et al.[4]

Note S2 IPTG Sensitivity observed in strain *Vn* NC8 despite constant *tfoX* expression

While it initially appeared to us that we had successfully restored the *lacI* sequence when creating *Vn* NC8 (Sanger sequencing had shown that missing base pairs at the end of the coding region were restored), subsequent whole genome sequencing showed that a novel 233 bp deletion within *lacI* arose during this new editing attempt (see Supplementary Figure S1). Throughout our attempts to edit and insert the combined *lacI/tfoX* inducible construct, there appears to have been consistent selection for defective *lacI* sequences which arise as a rare event during editing. The mechanism for this remains unclear. Despite the fact that strain *Vn* NC8 is insensitive to IPTG induction and continues to constitutively remain competent (Figure S7), with constant genomic expression of *tfoX* as a function of added IPTG (Figure S10), it exhibits dramatic sensitivity to IPTG addition. Falling total survival (in MCM) as a function of IPTG added spans two orders of magnitude (Figure S9), resulting in poor transformation yields despite minimal effects

to transformation frequency. IPTG-driven circuit toxicity could make a lot of sense if an excessively-strong Ptac promoter was causing excess tfoX expression. However, this is clearly not the case (Figure S10), especially given that from pMMB67EH-tfoX *tfoX* can be expressed by an additional order of magnitude.

While we have measured this effect most completely in strain NC8, we have anecdotally observed unanticipated IPTG sensitivity in other constructs as well when they are grown in MCM (in general, IPTG does not seem to cause issues in rich media). Ultimately, this represents a major issue in our strains, as for practical reasons it would be best to be able to turn competence on and off. We suspect that this phenomenon must somehow relate to our challenges creating strains with genomically-integrated *Vc tfoX* expression, but the mechanism for LacI/IPTG toxicity remains unclear. In future work we will pursue using alternative inducible promoters to see if we can fix the inducibility issue. However, since there may be limited gains to further overproducing *tfoX*, going forward we will also be pursuing alternative routes toward dysregulating natural competence.

Note S3 NPT protocol optimized for high efficiency

1. Prepare: LBv2 plates[1] with and without the appropriate antibiotic; 60% sterile glycerol; liquid nitrogen (if flash freezing);

2. Prepare 1× minimal competence media (MCM): 9 mM HEPES, 3 mM sodium acetate, 1.9 mM ammonium chloride, 1.6mM potassium phosphate, 7 mM potassium chloride, 1 mM magnesium sulfate, 31 mM magnesium chloride, and 350 mM sodium chloride. In order to prevent precipitation, 1 mL of undilute hydrochloric acid is used to lower the pH of 900 mL of deionized water prior to adding the media components and water to a total volume of 1 L. The final mixture is then gradually adjusted upwards to pH 7.4 using 1 M sodium hydroxide and sterile filtered. The media will precipitate if autoclaved.

3. Streak cells from a glycerol stock onto LBv2 plates for single colonies. Incubate overnight at 37 °C.

4. From a single colony, inoculate 20 mL of MCM in a sterile flask. Incubate for 18 hours, statically, at 30 °C. Note that MCM, and acetate in general, is a very poor nutrient, and growth will be only barely visible with the naked eye and not detectable with an OD meter.

5. Briefly resuspend cells by swishing the flask, and take as many 350 μL aliquots as needed. These can either be added to 110 μL 60% glycerol, flash frozen, and stored at -80 °C, or used immediately in subsequent steps.

6. If using frozen cells, thaw them at room temperature for $\approx$ 5 minutes.

7. Add $\geq$ 25 ng of plasmid DNA to the cells. Invert or vortex briefly to mix.

8. Allow cells to incubate statically for at least 45 minutes at 37 °C. Per Figure 3G, cells can be incubated for up to 3 hours with minimal additional gains to transformation frequency or yield at temperatures ranging from 20 to 37 °C.

9. Dilute in MCM as necessary to get single colonies (for a typical transformation, 1-2 orders of magnitude is sufficient), spread onto a prewarmed LBv2 plate, and grow at 37 °C for single colonies. Small colonies are visible 6-7 hours after plating. Efficiency is reduced if cells are diluted in a medium other than MCM.

Note S4 Room temperature NPT protocol optimized for no capital equipment

1. Prepare: LBv2 plates[1] with and without the appropriate antibiotic; 60% sterile glycerol.

2. Prepare 1× minimal competence media (MCM): 9 mM HEPES, 3 mM sodium acetate, 1.9 mM ammonium chloride, 1.6 mM potassium phosphate, 7 mM potassium chloride, 1 mM magnesium sulfate, 31 mM magnesium chloride, and 350 mM sodium chloride. In order to prevent precipitation, 1 mL of undilute hydrochloric acid is used to lower the pH of 900 mL of deionized water prior to adding the media

components and water to a total volume of 1 L. The final mixture is then gradually adjusted upwards to pH 7.4 using 1 M sodium hydroxide and sterile filtered. The media will precipitate if autoclaved.

3. Streak cells from a glycerol stock onto LBv2 plates for single colonies. From this point on, no capital equipment is necessary. When grown at room temperature, single colonies will become visible 24 hours after being struck out.

4. From a single colony, inoculate 20 mL of MCM in a sterile flask. Incubate statically at room temperature for 24 hours (although competency is maintained for at least up to 50, Main Figure 2E). Note that MCM, and acetate in general, is a very poor nutrient, and growth will be only barely visible with the naked eye and not detectable with an OD meter.

5. Briefly resuspend cells by swishing the flask, and take as many 350 μL aliquots as needed.

6. Add $\geq$ 25 ng of plasmid DNA to the cells. Invert to mix.

7. Allow cells to incubate statically for at least 45 minutes at room temperature. Spread on a room temperature LBv2 plate. Small colonies are visible 24 hours after plating at room temperature.

Supplementary Figures

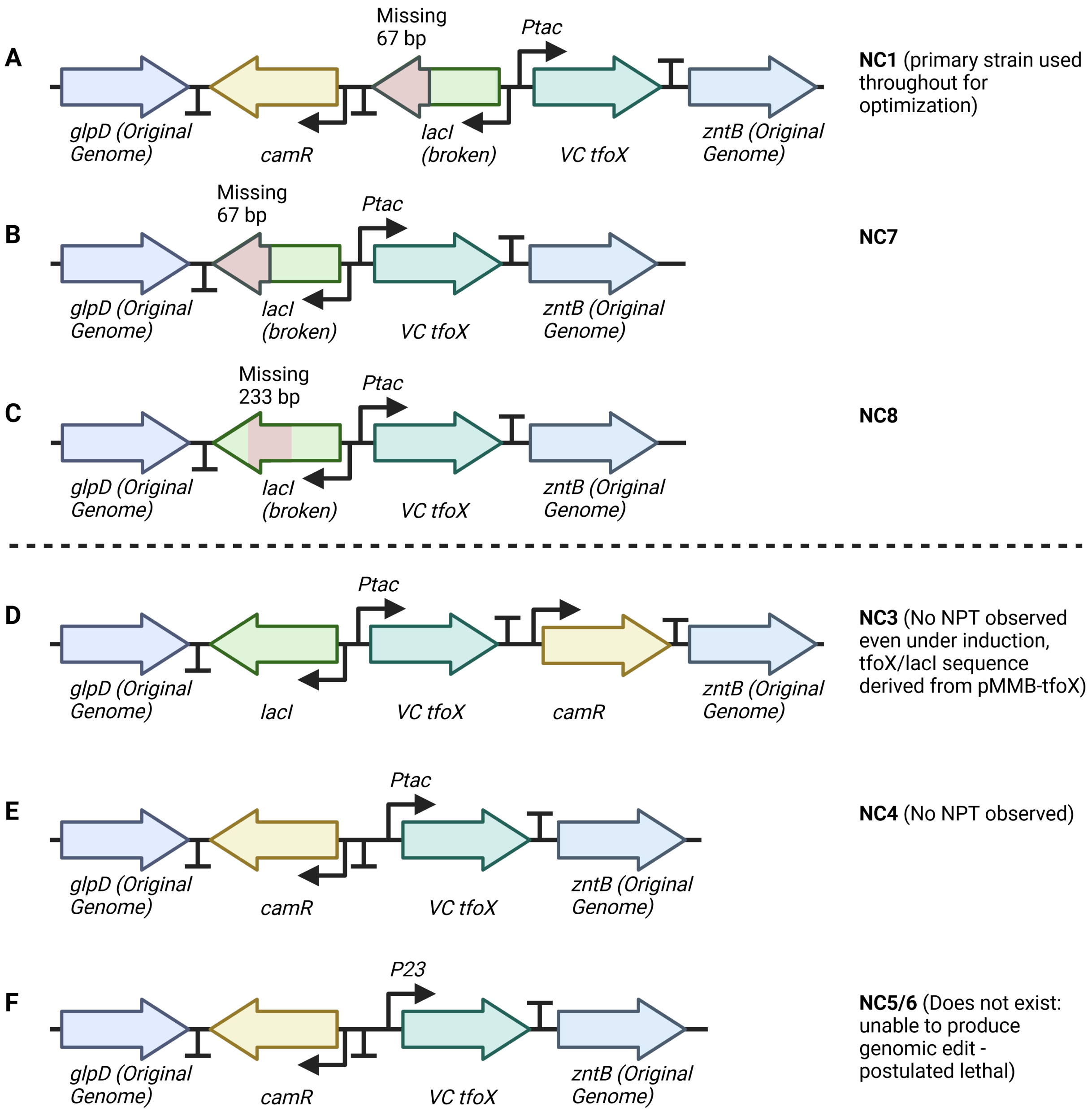

Supplementary Figure S1: **Schematics depicting the contextual arrangement of *Vc tfoX* insertion.** All iterations in this study were inserted at the same site in *V. natriegens* chromosome 1, downstream of the gene *glpD* and upstream of *zntB*. (**A**,**B**,**C**) 3 working designs for genomic *Vc tfoX* expression derived from the expression sequence in pST_140_LVL2 cam.[29] However, none of these exhibit inducibility of natural competence as *tfoX* expression is constitutively on, as could be expected given the defective *lacI* sequences. See Figure S10. (**D**,**E**,**F**) 4 failed designs for genomic expression of *tfoX*. In *Vn* NC3, the sequence which is inducible in PMMB67EH-tfoX[14] is inserted into the same site, but no NPT is observed even under IPTG induction. In *Vn* NC4, we sought to embrace the constitutive nature of *Vn* NC1 and simply delete the broken *lacI* sequence, but the resulting strain exhibited no NPT. A subsequent two variants (*Vn* NC5, NC6) based on the strong P23 promoter validated in the *V. natriegens* genome[13] could not be inserted into the genome successfully. Both versions use the same P23 promoter but in *Vn* NC6 an excessive second ribosomal binding site is deleted. All sequences for plasmids used to generate tDNA for genomic editing are included in Supplementary Information.

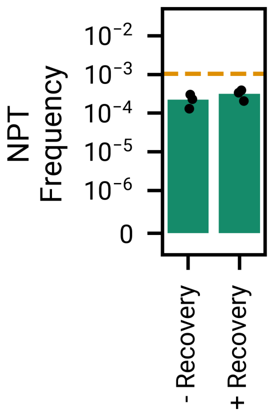

Supplementary Figure S2: **Strain *Vn* NC1 exhibits NPT when transformed using the protocol described in Dalia et al.** Here, we use *Vn* NC1 in the protocol as described in Reference 14 in lieu of MCM and our associated protocol. When NC1 is grown out in rich LBv2 media and then diluted into artificial seawater in the presence of transforming plasmid pDS5.30, we observe NPT at a frequency comparable to our protocol without flash freezing (Figure 3E). As we observe with our protocol (Figure 3A), use of a secondary recovery step does not increase the frequency of transformation. NPT frequency is shown relative to the no recovery condition used as a benchmark established in Figure 3 (dashed orange line).

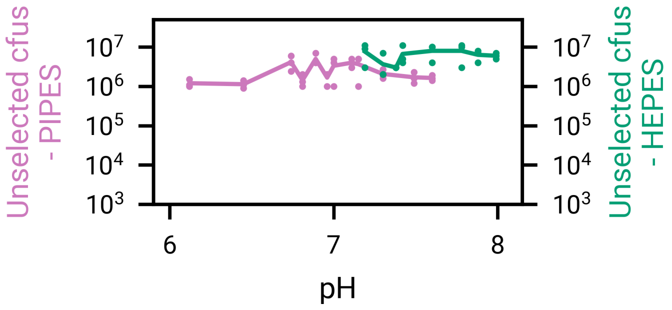

Supplementary Figure S3: **Total survivorship of *Vn* NC1 during transformation is slightly increased under higher pH in HEPES buffer.** Total number of unselected colony forming units in 50 μL of MCM after transformation, as a function of pH, buffered with either PIPES or HEPES, as indicated. *V. natriegens* readily grows in MCM from at least pH 6.12 to 7.99. Despite this, NPT is pH-dependent (Figure 2C), occurring at a reduced frequency for lower pH, and is undetectable at pH 6.12.

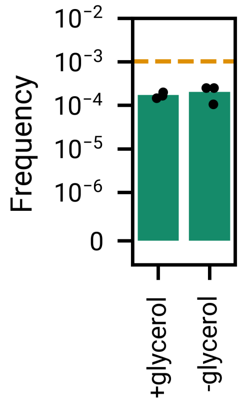

Supplementary Figure S4: **The addition of glycerol in immediate transformation of *Vn* NC1 after 30 °C outgrowth does not restore the transformation frequency observed in the case of flash freezing.** This indicates that the addition of glycerol is not the driver of increased transformation frequency. NPT frequency is shown relative to the no recovery condition used as a benchmark established in Figure 3 (dashed orange line).

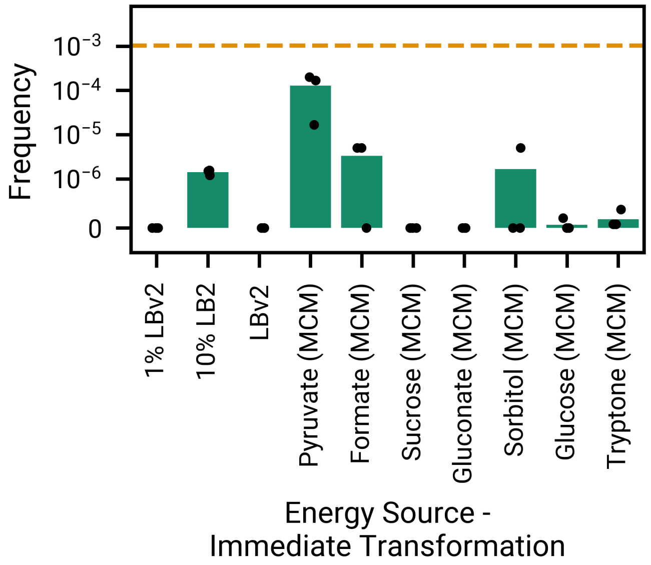

Supplementary Figure S5: **Pyruvate is an effective alternative carbon/energy source in lieu of acetate.** Under immediate transformation after outgrowth (with no flash freezing and -80 °C storage), pyruvate is the next-best carbon/energy source in lieu of acetate for driving natural transformation of *Vn* NC1 using MCM. All carbon/energy sources in MCM are at 3 mM. Additionally, a mixture of 10% LBv2 and 90% Instant Ocean Media produces measurable transformation. NPT frequency is shown relative to the no recovery condition used as a benchmark established in Figure 3 (dashed orange line).

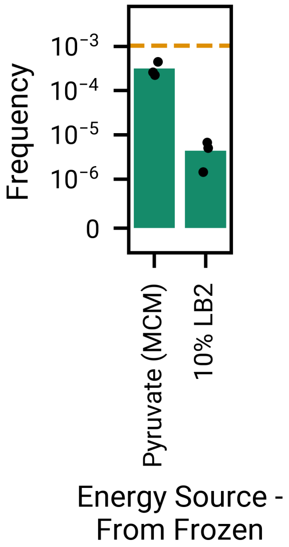

Supplementary Figure S6: ***Vn* NC1 grown out in Pyruvate MCM and 10% LBv2 can be frozen and preserved in a natural competence state.** As in the primary experiments with acetate-based MCM, freezing and -80 °C storage prior to transformation enhances transformation in pyruvate MCM and 10% LBv2 (compare with Figure S5). NPT frequency is shown relative to the no recovery condition used as a benchmark established in Figure 3 (dashed orange line).

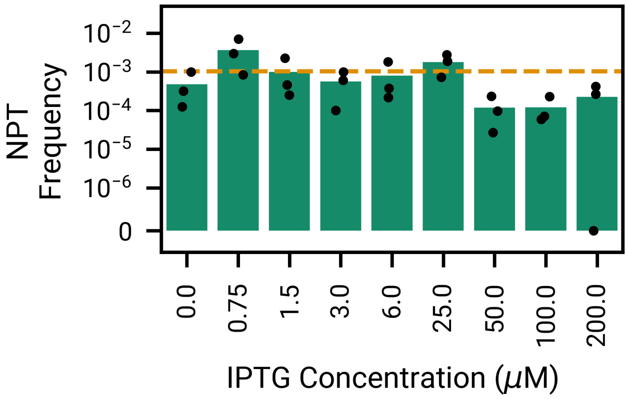

Supplementary Figure S7: ***Vn* NC8 remains in a constitutive natural competence state despite attempted correction of the *lacI* sequence.** This is bourne out by subsequent RT-qPCR measurements of *tfoX* expression levels (Figure S10) and reflective of a novel 233 bp deletion in *lacI*. NPT frequency is shown relative to the no recovery condition used as a benchmark established in Figure 3 (dashed orange line).

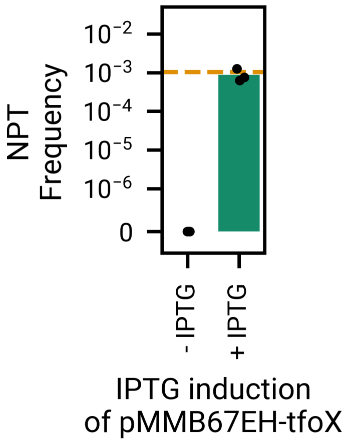

Supplementary Figure S8: **IPTG induction of pMMB67EH-tfoX[14] achieves the same frequency of NPT as strain NC1.** In this experiment, we use the same high efficiency protocol (with flash freezing) that we developed for our strain *Vn* NC1. There does not appear to be an increase in NPT frequency, despite an additional order of magnitude increase in *Vc tfoX* mRNA expression levels (Figure S10), indicating that there could be a ceiling to *tfoX* expression efficacy. As discussed in the main text, however, IPTG is only 50 μM, half of what is typically used for induction. NPT frequency is shown relative to the no recovery condition used as a benchmark established in Figure 3 (dashed orange line).

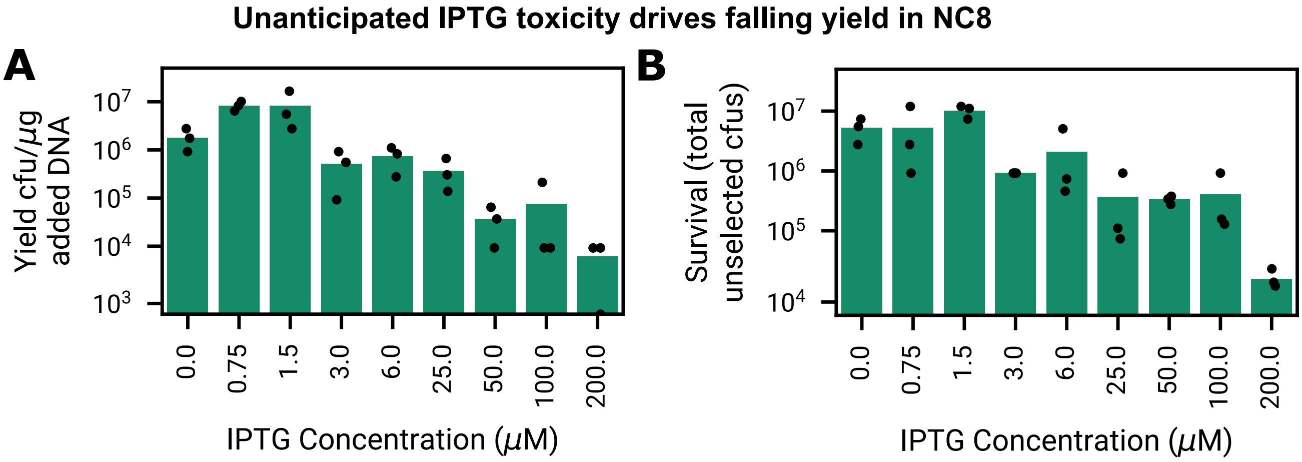

Supplementary Figure S9: **Despite the lack of inducibility of strain *Vn* NC8, it is sensitive to addition of IPTG with falling total survival in MCM.** While frequency of NPT stays largely constant (Figure S7), the yield (**A**) falls as a result of falling total survival (**B**). Since transcriptional expression of *tfoX* (Figure S10) remains constant, and we have been unsuccessful in our attempts to create a version with the correct *lacI* sequence (Supplementary Note S2), it is unclear what the mechanism for this toxicity may be.

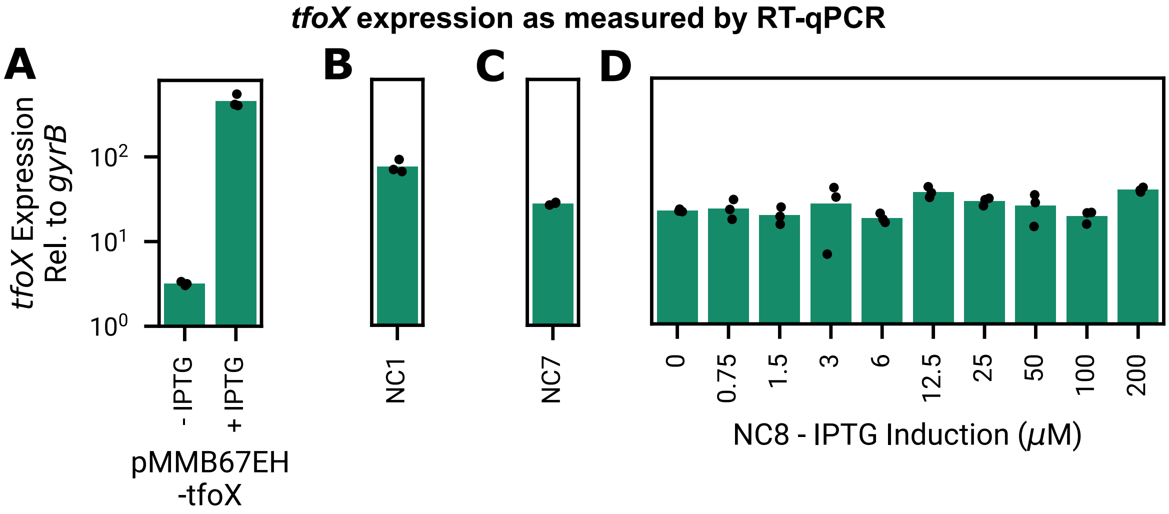

Supplementary Figure S10: **RT-qPCR establishes transcriptional upper and lower bounds for *Vc tfoX* expression necessary to trigger NPT in *V. natriegens*.** (**A**) Induction of strain Δ*dns* containing plasmid pMMB67EH-tfoX[14] exhibits IPTG-inducible *Vc tfoX* expression, as expected. Further, while leaked *tfoX* transcripts are measured, they appear to be tolerated as no leaky transformation in the - IPTG condition is observed (Figure S8). (**B**,**C**) Constitutive expression of *tfoX* mRNA from strain NC1 is higher than that of NC7. (**D**) Corresponding to the lack of IPTG inducibility shown in Figure S7, mRNA production from the Ptac promoter in NC8 remains insensitive to the addition of IPTG. In all RT-qPCR experiments in this figure, mRNA is extracted from cells grown out in MCM as they would prior to the addition of transforming DNA. Measured *tfoX* is normalized relative to expression of genomic *gyrB* mRNA expression.
